# Fermentation of *Vaccinium floribundum* Berries with *Lactiplantibacillus plantarum* Reduces Oxidative Stress in Endothelial Cells and Modulates Macrophages Function

**DOI:** 10.3390/nu14081560

**Published:** 2022-04-08

**Authors:** Luisa Marracino, Angela Punzo, Paolo Severi, Rosane Nganwouo Tchoutang, Celia Vargas-De-la-Cruz, Francesca Fortini, Francesco Vieceli Dalla Sega, Alessia Silla, Emanuele Porru, Patrizia Simoni, Valentina Rosta, Alessandro Trentini, Achille Wilfred Ouambo Talla, Silvana Hrelia, Carlo Cervellati, Paola Rizzo, Cristiana Caliceti

**Affiliations:** 1 Department of Translational Medicine and Laboratory for Technologies of Advanced Therapies (LTTA), University of Ferrara, 44121 Ferrara, Italy; svrpla@unife.it (P.S.); nganwouorosane@gmail.com (R.N.T.); achillewilfred.ouambotalla@unife.it (A.W.O.T.); rzzpla@unife.it (P.R.); 2 Department of Chemistry “Giacomo Ciamician” Alma Mater Studiorum, University of Bologna, 40126 Bologna, Italy; angela.punzo2@unibo.it; 3 Department of Pharmacology, Bromatology and Toxicology, Faculty of Pharmacy and Biochemistry, Universidad Nacional Mayor de San Marcos, Lima 15081, Peru; cvargasd@unmsm.edu.pe; 4 E-Health Research Center, Universidad de Ciencias y Humanidades, Lima 15314, Peru; 5 Maria Cecilia Hospital, GVM Care & Research, 48033 Cotignola, Italy; ffortini@gvmnet.it (F.F.); fvieceli@gvmnet.it (F.V.D.S.); 6 Department for Life Quality Studies, Alma Mater Studiorum, University of Bologna, 40126 Bologna, Italy; alessia.silla2@unibo.it (A.S.); silvana.hrelia@unibo.it (S.H.); 7 Department of Medical and Surgical Sciences, Alma Mater Studiorum, University of Bologna, 40138 Bologna, Italy; emanuele.porru2@unibo.it (E.P.); patrizia.simoni@unibo.it (P.S.); 8 Biostructures and Biosystems National Institute (INBB), 00136 Rome, Italy; 9 Department of Biomedical and Specialist Surgical Sciences, University of Ferrara, 44121 Ferrara, Italy; valentina.rosta@unife.it (V.R.); trnlsn@unife.it (A.T.); crvcrl@unife.it (C.C.); 10 Department of Biomedical and Neuromotor Sciences, Alma Mater Studiorum, University of Bologna, 40126 Bologna, Italy; 11 Interdepartmental Centre for Renewable Sources, Environment, Sea and Energy (CIRI FRAME), Alma Mater Studiorum, University of Bologna, 40126 Bologna, Italy

**Keywords:** lactic acid bacteria (LAB), fermentation, Pushgay (*Vaccinium floribundum*) berries, antioxidant activity, endothelial dysfunction, immunostimulant activity

## Abstract

Accumulating evidence suggests that high consumption of natural antioxidants promotes health by reducing oxidative stress and, thus, the risk of developing cardiovascular diseases. Similarly, fermentation of natural compounds with lactic acid bacteria (LAB), such as *Lactiplantibacillus plantarum*, enhances their beneficial properties as regulators of the immune, digestive, and cardiovascular system. We investigated the effects of fermentation with *Lactiplantibacillus plantarum* on the antioxidant and immunomodulatory effects of Pushgay berries (*Vaccinium floribundum*, Ericaceae family) in human umbilical vein endothelial cells (HUVECs) and macrophage cell line RAW264.7. Polyphenol content was assayed by Folin–Ciocalteu and HPLC-MS/MS analysis. The effects of berries solutions on cell viability or proliferation were assessed by WST8 (2-(2-methoxy-4-nitrophenyl)-3-(4-nitrophenyl)-5-(2,4-disulfophenyl)-2H-tetrazolium, monosodium salt and Lactate dehydrogenase (LDH) release, Trypan blue exclusion test, and Alamar blue assay. Antioxidant activity was evaluated by a cell-based chemiluminescent probe for the detection of intracellular H_2_O_2_ production in HUVECs. Heme oxygenase-1 (HO-1) expression levels were investigated by RT-qPCR. Glutathione reductase (GR), glutathione peroxidase (Gpx), superoxide dismutase (SOD), and catalase (CAT) activities, as markers of intracellular antioxidant defense, were evaluated by spectrophotometric analysis. The immunomodulatory activity was examined in RAW 264.7 by quantification of inducible nitric oxide synthase (iNOS) and Tumor Necrosis Factor—alpha (TNFα) by RT-qPCR. Data showed that fermentation of Pushgay berries (*i*) enhances the content of quercetin aglycone, and (*ii*) increases their intracellular antioxidant activity, as indicated by the reduction in H_2_O_2_-induced cell death and the decrease in H_2_O_2_-induced HO-1 gene expression in HUVECs treated for 24 h with fermented berries solution (10 µg/mL). Moreover, treatment with Pushgay berries for 72 h (10 µg/mL) promotes cells growth in RAW 264.7, and only fermented Pushgay berries increase the expression of iNOS in the same cell line. Taken together, our results show that LAB fermentation of Pushgay berries enhances their antioxidant and immunomodulatory properties.

## 1. Introduction

Oxidative stress is involved in the development of several diseases, including atherosclerosis, the major cause of many cardiovascular diseases (CVDs) [1]. The molecular mechanisms by which oxidative stress contributes to atherosclerosis are well characterized [2,3,4]. Specifically, the dysregulated production of intracellular reactive oxygen species (ROS) causes phenotypical changes to the endothelial cell surface leading to endothelium dysfunction, which precedes the formation of atherosclerotic plaque [1]. Thus, the maintenance of the endothelium function represents a promising approach to prevent CVDs. 

In recent years, the potential role of natural bioactive compounds in preventing and treating disorders where oxidative stress is involved, such as cancers, metabolic diseases (e.g., obesity and diabetes), neurodegenerative, and CVDs, has clearly emerged. Indeed, since natural products exert beneficial biological activities and possess pharmacological properties, they could play a valuable role in drug discovery [5,6,7].

Epidemiological and clinical studies have reported the beneficial properties of berries in terms of natural antioxidant capabilities and decreased risk of CVDs [8,9]. Berries are significantly enriched in flavonoids and phenolic acids, which are responsible for their protective effects, such as reducing endothelial dysfunction [10] and reducing oxidative stress, and mitochondrial damage [11,12], as well as the anti-inflammatory mediators induction [13]. Both in vivo and in vitro studies have shown that flavonoids, particularly quercetin and its metabolites, dampen oxidative stress, inflammation, and interfere with atherosclerosis progression [14,15,16,17,18,19,20,21].

Therefore, it is not surprising that berries have been increasingly employed worldwide as components of functional food and dietary supplements. In this regard, there is growing interest in studying the beneficial effects of berries of the *Vaccinium* species from South America, such as bilberry or blueberry, on human health [22]. *Vaccinium floribundum*, commonly known as Pushgay, Mortiño, or Andean blueberry, is a deciduous, spreading shrub, similar to European blueberries, that belongs to the family Ericaceae. This species is native to Ecuador and Peru but is also found in other countries of South and Central America, where it grows at altitudes from 1800 to 3800 m [23]. Local people widely consume these berries as fresh fruit or processed products. Moreover, local communities use the extracts of this plant to treat many pathologies, including diabetes and inflammation [24]. Pushgay berries are rich in quercetin, hydroxycinnamic acids and anthocyanins [25] responsible for their anti-oxidant, anti-inflammatory, and anti-microbial properties [26]. However, the poor bioavailability of the polyphenols present in the berries, mostly influenced by their complex chemical structures, points out their direct effect on human health. None of the studies conducted so far have investigated the potential enhancement of the beneficial effects of Pushgay berries after fermentation.

Traditionally, fermentation has been used to preserve perishable foods. However, this process has attracted significant attention since it leads to the production of health-promoting components that increase the nutritional value of foods. Indeed, the fermentation increases the bioavailability of polyphenols [27], since enzymes present in lactic acid bacteria (LAB) produce changes in both profile and types of bioactive compounds, producing simpler phenolic compounds that may be absorbed in the duodenum. [28,29].

LAB plays an important role not only in improving the antioxidant properties of different foods but also in modulating immune response. Some LAB strains (mainly *Lactobacillus* and *Streptoccoccus*) and *Bifidobacterium* modulate the innate and adaptive immune response through the induction of pro-inflammatory cytokines, such as TNFα, interferon-γ (IFN-γ), some interleukins (IL) (IL-1β, IL-6, IL-12) and nitric oxide (NO) [30]. Several studies have shown that LAB-mediated immunomodulation is species-specific: indeed, some strains of *Lactobacillus* reduce the expression levels of pro-inflammatory mediators while others exert opposite effects [31].

This study aimed to evaluate the phytochemical profile and the antioxidant and immunomodulatory activities on endothelial cells and macrophages of Pushgay berries before and after a fermentation process with *Lactiplantibacillus plantarum.*

We show that fermentation of Pushgay berries enhances their immunostimulant and antioxidant performance, thus supporting existing studies on the contribution of lactic bacteria fermentation to the health benefits of food. 

## 2. Materials and Methods

### 2.1. Reagents

Human umbilical vein endothelial cells (HUVECs), Dulbecco’s Modified Eagle’s Medium (DMEM), basal medium M200, IFN-γ (100 U/mL in PBS), TNFα (10 μg/mL in Milli-Q H_2_O), Superscript III reverse transcriptase, random primers, dNTPs, and RNaseOut were obtained from Life Technologies (Carlsband, CA, USA). RAW264.7 were obtained from ATCC (Manassas, VA, USA). Fetal bovine serum (FBS) and EGM-2 Endothelial Medium SingleQuot kit were purchased from Lonza (Basel, Switzerland). RNeasy kit for RNA extraction was purchased from Qiagen (Hilden, Germany). Perfecta SYBR Green Supermix for Quantitative RT-PCR was obtained from Quanta Biosciences (Gaithersburg, MD, USA). Oligonucleotides for qRT-PCR were purchased from IDT (Coralville, IA, USA). H_2_O_2_-CL-510 probe (AquaSpark™ Peroxide Probe) was provided by Biosynth (Staad, Switzerland). The Cytotoxicity LDH Assay kit and the Cell Counting Kit-8 were purchased from Dojindo Molecular Technologies (Kumamoto, Japan). *Lactiplantibacillus plantarum* starters were supplied by the American Type Culture Collection (ATCC, Manassas, VA, USA). De Man, Rogosa, and Sharpe (MRS) agar powder and yeast from *Saccharomyces cerevisiae* were purchased from Sigma-Aldrich (St. Louis, MO, USA). Anthocyanins and flavonols standards for HPLC-ESI-MS/MS analysis were bought from Carbosynth (USA, Canada & South America). Protease inhibitor mix was purchased from SERVA Electrophoresis GmbH (Heidelberg, Germany) and phosphatase inhibitor mix (phosSTOP) from F. Hoffmann–La Roche SA (Basel, Switzerland), The other materials were purchased from Sigma-Aldrich.

### 2.2. Pushgay Berries Fermentation

Pushgay berries were collected between March and April 2019 in Cajamarca province (Cajamarca, Perú) and their taxonomy was certified by the Herbario San Marcos (National University of San Marcos, Lima, Peru) at the National University of San Marcos (Lima, Peru). Berries were crushed, freeze-dried by a Heto PowerDry LL1500 (Thermo Fisher Scientific, Waltham, MA, USA) finely ground in a mortar, and stored at −20 °C until use [32]. 

For the fermentation process, *Lactiplantibacillus plantarum* at 5% was grown in 10 mL of MRS agar broth for 16 h at 30 °C under anaerobic conditions, then diluted to 1% with an additional 40 mL MRS agar broth, and allowed to grow for additional 16 h under the same conditions. 200 g of frozen berries were disinfected with sodium hypochlorite 0.5% (*w*/*v*), solubilized in 500 mL of distilled water, and placed in a dark bottle. The solution was exposed to a heat shock at 70 °C for 10 min, then cooled in an ice bath until it reached a temperature of 40 °C. Finally, they were heated to 85 °C for 5 min and cooled in an ice bath until they reached a temperature of 25 °C. The solution was divided into sterile flasks, then the inoculum (5 × 10^7^ CFU/mL) and a yeast extract were added (0.01 and 0.4% *w*/*v*, respectively). Fermentation was carried out at a temperature of 30 °C for 48 h, in the dark and under constant stirring. The fermentates were lyophilized and stored at −20 °C before being shipped to Italy.

Not fermented Pushgay berries were just crushed, freeze-dried by a Heto PowerDry LL1500 (Thermo Fisher), finely ground in a mortar, lyophilized, and stored at −20 °C before being shipped to the laboratories at the University of Bologna, Italy.

### 2.3. Preparation of Pushgay Berries Solutions

Once in Bologna (Italy), 10 mg of lyophilized Pushgay berries (fermented or not) were solubilized in 1 mL of sterile dimethyl sulfoxide (DMSO) using a sonicator (Soniprep 150 Ultrasonic Disintegrator). Specifically, five sonication cycles were performed while keeping the samples on ice and in the dark, vortexing the samples between the cycles. The solutions obtained was subsequently aliquoted and stored at −20 °C. Samples were briefly centrifuged at a low speed (700× *g*) before cell treatment.

### 2.4. Evaluation of Total Phenolic Content

The total phenolic content of lyophilized berries was determined using the Folin–Ciocalteu reagent method described by Ainsworth et al. [33].

Briefly, 100 µL of fermented or non-fermented berries solutions were mixed with a double volume of Folin–Ciocalteu reagent. Then, 800 µL of 700 mM aqueous sodium carbonate was added, and the reaction mixture was incubated for 2 h at room temperature. After the incubation, 200 µL of each sample were transferred into a 96-well microplate. The absorbance was spectrophotometrically read at 765 nm using distilled water as a blank. Gallic acid (range 100–1000 μg/mL) was used as standard, and the total phenolic content was expressed as µg of gallic acid equivalent (GAE)/µg lyophilized berry.

### 2.5. HPLC-ESI-MS/MS Analysis

Liquid chromatography was performed using a 2690 Alliance system (Waters, Milford, MA, USA). Analytical separation was performed using Phenyl-Hexyl 1.7 µm, 150 mm × 2.1 mm i.d. (Waters, Milford, MA, USA). The mobile phase was constituted as follows: 15 mM ammonium acetate in water adjusted to pH 8.0 with ammonia (solvent A) and methanol 99.9% (solvent B). Chromatographic separation was achieved at a 0.15-mL/min flow rate under gradient elution conditions: 95% A for 5 min, 95–40% A from 5 to 15 min, 40–20% from 15 to 20 min, 20% A from 20 to 25 min, 20–95% A from 25 to 27 min, and 95% A from 27 to 35 min. All the changes in the mobile phase composition were linear. The analytical column was maintained at 30 °C. The column effluent was introduced into the ESI source, operating in positive ionization mode, connected to a triple quadruple mass spectrometer (Quattro-LC, Micromass) operating in the multiple reaction monitoring (MRM) acquisition mode. Standard solutions of 3 anthocyanins were used to evaluate polyphenols profile of fermented and non-fermented Pushgay berries: malvidin-3-O-glucoside (MAL), quercetin-3-Oglucoside (QUE), and quercetin aglycone (QA). The most abundant signal for each compound in multiple reaction monitoring mode (MRM) was monitored for the quantification [*m*/*z* 330.9 → 330.9 (quercetin, aglycone), *m*/*z* 302.9 → 302.9 (quercetin-O-glucoside), *m*/*z* 330.9 → 330.9 (malvidin-O-glucoside), and *m*/*z* 153.6 → 125.6 (hydroxytyrosol)]. The analytical method was developed and validated according to ICH guidelines to satisfy high analytical parameters in terms of accuracy and reproducibility. Limit of detection (LOD) and limit of quantification (LOQ) were determined by signal to noise ratio (LOD = 3, LOQ = 10). Recovery and the matrix effect were evaluated for each compound before the sample analysis. Seven point calibration curves (0.5, 1, 2.5, 5, 10, 25, and 50 ng/mL) were used for the quantification of each compound using standard solutions in the mobile phase (phase A:phase B 95:5). Internal standard hydroxytyrosol was used for calibration curves at a fixed concentration of 5 ng/mL. Then, 100 mg of each sample was extracted by sonication with 1 mL of 70% ethanol solution three times. The samples were centrifuged, the supernatants were pooled, and 10 ul of the sample was injected into the HPLC-MS.

### 2.6. Cell Culture

HUVECs were plated on 1.5% gelatin-coated tissue culture dishes and maintained in phenol red-free basal medium M200 containing 2% FBS and EGM-2 at 37 °C with 5% CO_2_. HUVECs at passages 2 to 7 were actively proliferating (70–90% of confluency) when they were harvested and analyzed. 

RAW 264.7 cells (murine monocyte/macrophage cell line) were cultured in DMEM without phenol red containing 4 mM L-glutamine, 4500 mg/L glucose, 10% FBS, 100 IU/mL penicillin, and 100 μg/mL streptomycin at 37 °C with 5% CO_2_; for experiments, cells were used at passages 2 to 7, to avoid possible changes of cellular phenotype.

### 2.7. Cell Viability and Proliferation Assays

In HUVECs, WST8 (2-(2-methoxy-4-nitrophenyl)-3-(4-nitrophenyl)-5-(2,4-disulfophenyl)-2H-tetrazolium, monosodium salt) was used to determine the cell viability. Indeed, in the presence of an electron mediator, WST8 is reduced to red formazan dye by dehydrogenases present in the cells. The amount of red dye generated is directly proportional to the number of living cells [34].

For the experiments, 1.0 × 10^4^ cells/well HUVECs were seeded in a 96-well plate. After 80% confluence had been reached, cells were treated with different concentrations of berries solutions: fermented Pushgay (FP) and Pushgay (P) in complete culture medium for 24 h (range 0.25–100 µg/mL). The decrease in absorbance between the 24 h treatment (representing t1) and the control (representing t0) was monitored at 37 °C at 450 nm using an AMR-100 Microplate reader (Allsheng, Hangzhou, China).

To determine cell viability in RAW 264.7, cells in the logarithmic growth phase were harvested and seeded in six well-plates (3.0 × 10^5^ cells/well) overnight. Cells were incubated for 24 h with different concentrations of berries solutions (range 2.5–5 and 10 μg/mL in 0.1% DMSO). At the end of incubation, viability was determined with the Burker chamber utilizing the Trypan Blue exclusion test.

The evaluation of the effects of berries on cell proliferation/metabolism in RAW 264.7 was investigated through the Trypan Blue and Alamar Blue assays. For Trypan Blue assay, RAW 264.7 cells, harvested in the logarithmic growth phase, were plated in triplicate in 24-well plates (3.0 × 10^4^ cells/well) overnight. Subsequently, cells were grown at 37 °C, 5% CO_2_ for 24 h and treated with 10 μg/mL of each berry solution and further incubated for 24 and 72 h. At the end of incubation, cell number was determined with the Burker chamber after staining with Trypan Blue. 

For the Alamar Blue assay, RAW 264.7 cells in the logarithmic growth phase were harvested and plated in 24 wells in triplicate (3.0 × 10^4^ cells/well) overnight. Next, cells were grown at 37 °C, 5% CO_2_ for 24 h and incubated with 10 μg/mL of each berry solution for 24 and 72 h before adding Alamar Blue solution (5%) in complete cell culture medium. Cells were incubated at 37 °C, 5% CO_2_ for 3 h. Next, 100 μL of the medium was transferred from each well to a 96-well plate. The reading was carried out with a spectrophotometer at 570 nm (Thermo Electron Corp., model MultiskanEX, Vantaa, Finland).

### 2.8. Cytotoxicity

In HUVECs, the toxicity of the berries solutions was assessed by lactate dehydrogenase (LDH) release. The enzyme LDH catalyzes the conversion of lactate to pyruvate via NAD+ reduction to NADH; next the diaphorase reduces tetrazolium salt, oxidizing NADH, to a red formazan product that can be spectrophotometrically determined at 490 nm. LDH release from HUVECs treated for 24 h with berries solutions was monitored by collecting aliquots of the medium under the same experimental conditions performed above for cell viability assays, as previously reported [35]. The decrease in absorbance between the treatment after 24 h and the control was monitored at 37 °C at 490 nm using an AMR-100 Microplate reader (Allsheng).

### 2.9. RNA Extraction

HUVEC cells (1.5 × 10^5^ cells/well) were seeded in six well-plates and incubated with 10 μg/mL of berries solutions, in the presence or absence of H_2_O_2_ (300 µM), for 24 h. RAW 264.7 cells (4.0 × 10^5^ cells/well) were seeded in six well-plates and incubated with 10 μg/mL of berries solutions, in the presence or absence of IFN-γ 100 U/mL, for 24 h. Total RNA was extracted using the commercial kit RNeasy mini Kit (Qiagen, Hilden, Germany). The concentration and purity of RNA were determined using the Nanodrop 1000 spectrophotometer (Thermo Fisher Scientific).

### 2.10. Reverse Transcriptase-Quantitative PCR

Total RNA (500 ng) was reverse transcribed in a volume of 25 μL using 250 units of SuperScript III reverse transcriptase (Life Technologies, Carlsbad, CA, USA) and 50 ng of random hexamers using the reaction conditions described in [36]. Next, 2 μL of the cDNA solution were used for the quantitative, real time PCR experiments to measure the amount of transcripts. Real-time PCR reactions were performed on a 7500 Fast Real-Time PCR System (Applied Biosystems, Life Technologies, Carlsbad, CA, USA) using PerfeCta SYBR Green SuperMix with ROX kit (VWR International), according to the manufacturer’s protocol, in a final volume of 23 μL. Primers were purchased from IDT (Coralville, IA, USA). Differences in gene expression levels were determined by the 2^−ΔΔCt^ formula [37], using RPL13A (ribosomal protein L13-A) as the reference gene. HO1 forward 5′-CAACAAAGTGCAAGATTCTG-3′, reverse 5′-GATTCACATGGCATAAAG-3′; RPL13A forward 5′-CACCCTGGAGGAGAAGAGGA-3′, reverse 5′-CCGTAGCCTCATGAGCTGTT-3′.

iNOS forward 5′-GGCAGACTGGATTTGGCTGG-3′, reverse 5′-CAACATCTCCTGGTGGAACAC-3′; TNFα forward 5′-ACTGAACTTCGGGGTGATCG-3′, reverse 5′-CCACTTGGTGGTTTGTGAGTG-3′.

### 2.11. Antioxidant Activity of Berries Solutions

The intracellular antioxidant activity of the berries was estimated in HUVECs by using the chemiluminescent (CL) cell-based bioassay previously described [38].

Briefly, two days before the experiment, HUVECs (1.0 × 10^4^ cells/well) were plated in a 96-well black microtiter with clear bottom and on the next day, cells were treated with serial dilutions of FP and P (range 0.25–25 µg/mL) for 24 h. Then, cell medium was removed and 100 μL of the H_2_O_2_-CL probe working solution (10 μM of CL probe in PBS, pH 7.5, final concentration 5 μM) was added. After the incubation for 20 min at 37 °C, 100 µL of the oxidant agent menadione (final concentration 25 μM in PBS, pH 7.4) was dispensed in each well to induce intracellular H_2_O_2_ production. CL emission signal was monitored for 40 min using a Luminoskan™ Ascent luminometric plate reader. The temperature was maintained at 37 °C during the measurement and the dose–response curve was obtained by plotting the CL signal versus the actual concentration of the menadione and fitting the experimental data to a straight line using the method of least squares.

### 2.12. Preparation of Cell Lysates for Enzymatic Assay

HUVEC cells (1 × 10^6^ cells/dish) were seeded in a 100 mm Petri dish and incubated with 10 μg/mL of berries solutions, in the presence or absence of H_2_O_2_ (300 µM), for 24 h. The lysates were obtained by scraping cells in a chilled buffer (PBS containing 1 mM phenylmethanesulfonyl fluoride, protease and phosphatase inhibitors mix according to manufacturer’s instructions). The lysates were then incubated at +4 °C for 30 min and subsequently centrifuged at 10,000× *g* for 10 min. The protein concentration in the supernatant was determined according to Bradford method [39].

### 2.13. Glutathione Reductase (GR) Activity Assay

GR activity assay was performed according to Smith and collaborators [40]. The assay is based on the reduction in oxidized glutathione (glutathione disulfide, GSSG) to the reduced form (GSH) performed by GR. GSH, in turn, can react with DTNB [5,5′-dithiobis (2-nitrobenzoic acid)], increasing the absorbance at 412 nm. In brief, 10 μL of cell lysate were added to 240 μL reaction mix composed by 100 mM potassium phosphate buffer, pH 7.4 with 0.141 mM NADPH, 0.75 mM DTNB, and 1.41 mM GSSG. The increase in absorbance was spectrophotometrically monitored at 1 min intervals over 10 min. GR activity was calculated using extinction coefficient 14,150 M^−1^ cm^−1^ and expressed as units per milligram protein.

### 2.14. Glutathione Peroxidase (Gpx) Activity Assay

Gpx activity assay was performed by indirect spectrophotometric analysis according to the method of Engel et al. [41], based on the oxidation of GSH to GSSG performed by Gpx, and simultaneously reduced again to GSH by GR with the consumption of NADPH. Briefly, 5 μL of cell lysate was added to 145 μL of reaction mix (phosphate-buffered saline, pH 7.4 with 3 U/mL of GR, and 1 mM of GSH) and incubated at 37 °C for 10 min. Then, 100 µL of the second reaction mix (phosphate-buffered saline, pH 7.4 with 0.2 mM NADPH, and 0.4 mM tert-butyl hydroperoxide) were added to the reaction tube. The reduction in NADPH concentration was monitored spectrophotometrically at 340 nM at 30 s intervals over 20 min. Gpx activity was calculated using extinction coefficient 6220 M^−1^ cm^−1^ and expressed as units per milligram protein.

### 2.15. Catalase (CAT) Activity Assay

CAT assay was performed by indirect spectrophotometric analysis by measuring the absorbance of hydrogen peroxide consumed by CAT. Briefly, 20 μL of cell lysate were added to 230 μL of 50 mM sodium phosphate buffer, pH 7.0 with 12 mM H_2_O_2_. One unity of CAT is defined as 1 μmol of H_2_O_2_ consumed for minute. CAT activity is expressed as units per milligram protein.

### 2.16. Superoxide Dismutase (SOD) Activity Assay

SOD assay was performed according to the method of Cervellati et al. [42]. Briefly, 10 μL of cell lysate were added to 10 μL of 50 mM phosphate buffer, pH 7.8 with 0.2 U/L of xanthine oxidase. Then, 250 μL of reaction mix (50 mM phosphate buffer, pH 7.8 with 900 μM xanthine, and 85 μM cytochrome C) were added to the reaction tube. The rate of cytochrome C reduction was monitored spectrophotometrically at 550 nm at 10 s intervals over 5 min.

### 2.17. Statistical Analysis

Results are expressed as means of at least three independent experiments with SD. Differences between the means were determined by unpaired student’s t-test or one-way ANOVA, followed by Bonferroni multiple comparison test using the GraphPad Prism Software, version 6.0 (GraphPad Software, Inc., La Jolla, CA, USA). A *p*-value ≤ 0.05 is statistically significant. 

## 3. Results

### 3.1. Content of Polyphenols in Berries Solutions

The total phenolic content was determined by the Folin–Ciocalteu reagent method obtaining a similar polyphenols concentration in fermented and non-fermented Pushgay berries (P: 2.38 ± 0.01 and FP: 2.95 ± 0.14 µg GAE/µg lyophilized berry) (Table 1), in line with previous data [32]. 

Quercetin glycoconjugates are the major flavonoid compounds in Pushgay berries [24,25] and the analysis of the phenolic profile of FP and P was obtained through untargeted characterization by ultra-high-performance liquid chromatography coupled to high-resolution mass spectrometry (UHPLC-HRMS) [32]. Cerrato et al. [32] showed that glyco-conjugations were mostly hydrolyzed following fermentation, and a higher concentration of free flavonols was found, mainly quercetin (97% of the total flavonol content) [32]. In the untargeted analysis, Cerrato et al. also identified, for the first time, a large number of phenolic acids and their derivatives (≈145 compounds) in FP and P; furthermore, anthocyanins in P were almost completely degraded after fermentation in phenolic acids [32].

In this study, we performed a targeted/quantitative analysis of quercetin (both the aglycone and glycoconjugate forms) through HPLC-ESI MS/MS (Table 1). In agreement with published data [32], our results showed that quercetin-3-O-glucoside was present in both FP and P: 96 ± 0.1 μg/g and 720 ± 0.1 μg/g, respectively, while quercetin aglycone was identified only in the FP with a concentration of 1680 ± 0.2 μg/g. Moreover, in line with previous analysis [32], malvidin-3-O-glucoside was not detected, possibly due to differences in the environment of growth and climate with respect with other Vaccinium berries [25]. 

Quercetin aglycone significantly increases after fermentation, suggesting that LAB fermentation induces the hydrolysis of glucosides (Table 1). Quercetin-3-O-glucoside has a higher solubility than the aglycone and is rapidly absorbed through the intestinal sodium-glucose cotransporter, irrespective of the position of the glucose moiety [43]. Nevertheless, quercetin-3-O-glucoside biological activity is limited due to the presence of the sugar moiety. Specifically, the absence of the sugar molecule on the flavonoids appears to be important for their free radical scavenging and antioxidant activities [44,45] and, consistently, treatment with quercetin aglycone is more effective than quercetin-3-O-glucoside in the induction of antioxidant and detoxifying enzymes, such as heme oxygenase (HO-1), NAD(P)H quinone oxidoreductase (NQO-1), and gamma-glutamylcysteine synthetase (GCLC) through the Nrf2/ARE pathway [44].

### 3.2. Cytotoxicity of Berries Solutions in HUVECs

**Figure 1:**
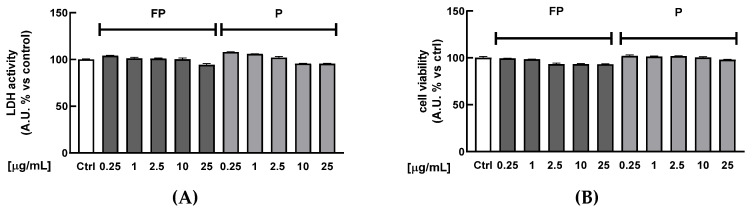
HUVECs were treated with fermented Pushgay (FP) or Pushgay (P) berries solutions for 24 h (concentration range: 0.25–25 µg/mL). (**A**) Cell viability was assessed by measuring red dye production at 490 nm (**B**) Cytotoxicity was quantified by spectrophotometrically measuring LDH released in cell medium. Ctrl (control, untreated cells); FP (cells treated with fermented Pushgay solution); P (cells treated with Pushgay solution).

HUVECs were treated for 24 h with FP or P solutions (between 0.25 and 25 µg/mL) to investigate their potential cytotoxicity. Figure 1A shows that treatment does not reduce the cell metabolism at any of the tested concentrations, as indicated by the absence of significant changes in the production of formazan dye in treated cells compared to the control. At the same time, lactate dehydrogenase (LDH) release was quantified in cell culture medium as a biomarker of cytotoxicity. Treatments with berries solutions for 24 h at any of the tested concentrations did not increase the release of LDH in cell medium (Figure 1B). Treatment with higher concentrations of FP and P solutions (50 and 100 μg/mL) significantly increased LDH release (data not shown), indicating cytotoxic effects at these concentrations. 

### 3.3. Effect of the Fermentation of Pushgay Berries on Antioxidant Activity in HUVECs

**Figure 2:**
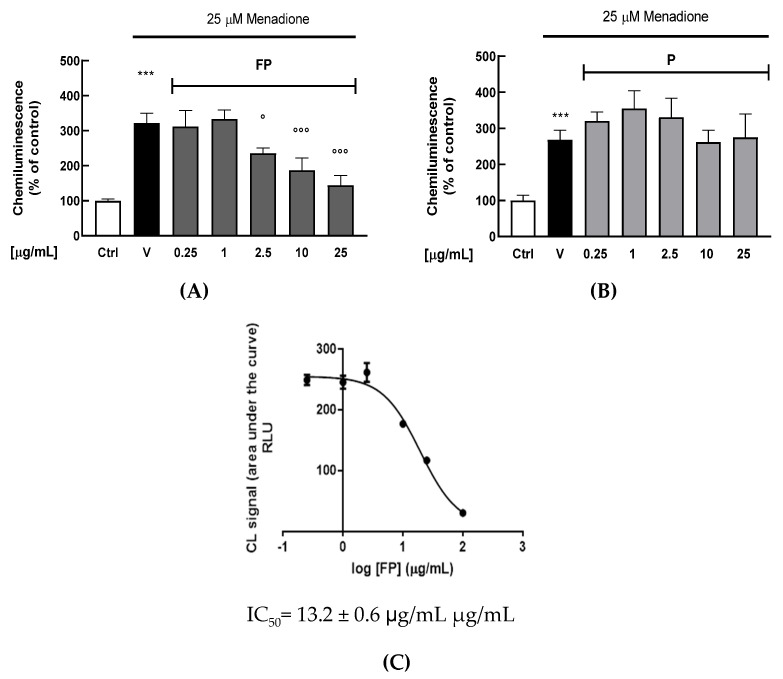
HUVECs were treated with fermented Pushgay (FP) (**A**) and Pushagay (P) (**B**) solutions for 24 h (concentrations range 0.25–25 µg/mL) and then exposed to the pro-oxidant agent menadione (25 μM for 40 min). Intracellular H_2_O_2_ production was measured for 40 min through the CL effect-based bioassay. (**C**) Concentration-response plot of inhibition of intracellular H_2_O_2_ production in HUVECs treated with FP (range 0.25–25 µg/mL). Results are expressed as mean ± SD of three independent experiments. *** *p* < 0. 001 significantly different from the ctrl. ° *p* < 0.05 and °°° *p* < 0.001 significantly different from V. Ctrl (control, untreated cells); V (control cells in presence of menadione); FP (cells treated with fermented Pushgay); P (cells treated with Pushgay).

A chemiluminescent (CL) cell-based bioassay was utilized to evaluate the intracellular antioxidant activity of FP or P solutions in HUVECs [38]. Briefly, HUVECs were treated with berries solutions for 24 h (range 0.25–25 μg/mL); the day of the experiment cell medium was changed, and cells were exposed to the oxidant agent menadione (25 μM). As shown in Figure 2A,B, FP significantly reduces intracellular H_2_O_2_ production at 2.5, 10 and 25 μg/mL (*p* < 0.05, *p* < 0.01 and *p* < 0.001, respectively), while treatment with *p* does not have any significant effect. The IC_50_ of FP was evaluated by a dose–response curve, obtaining a value of 13.2 ± 0.6 μg/mL (Figure 2C). 

### 3.4. Effect of Fermentation of Pushgay Berries on H_2_O_2_-Induced Cell Mortality in HUVECs

**Figure 3:**
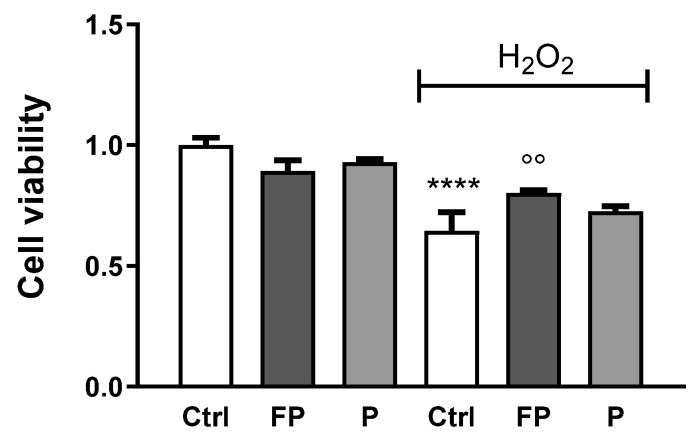
HUVECs were plated in six-well plates (1.5 × 10^5^ cells/well) and treated with 10 μg/mL of FP and P for 24 h followed by treatment with H_2_O_2_ (300 µM) for 24 h. Cell viability was determined by Trypan Blue staining. Results are expressed as mean ± SD of at least three experiments. **** *p* < 0.0001 significantly different from the control (ctrl). °° *p* < 0.01, significantly different from H_2_O_2_-treated HUVECs. Ctrl (control, untreated cells); H_2_O_2_ (cells treated with hydrogen peroxide); FP (cells pretreated with fermented Pushgay); P (cells pretreated with Pushgay).

Trypan Blue was used to determine the viability of HUVECs incubated with H_2_O_2_ (300 µM) for 24 h, following treatment with FP and P solutions (10 μg/mL) for 24 h. As expected, treatment with H_2_O_2_ reduced cell viability of HUVECs and FP, but not P, significantly counteracts this effect (Figure 3).

### 3.5. Effect of Fermentation of Pushgay Berries on HO-1 Expression in H_2_O_2_-Treated HUVECs

**Figure 4:**
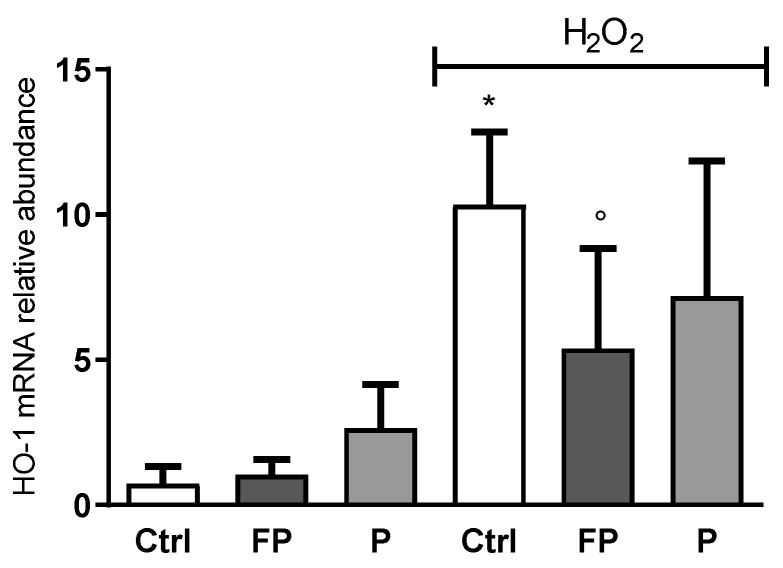
HUVECs were plated in 6-well plates (1.5 × 10^5^ cells/well) and treated with 10 μg/mL of FP and P for 24 h followed by injury with H_2_O_2_ (300 µM) for 24 h. HO-1 gene expression was assessed using qRT-PCR analysis. Gene expression levels were calculated using the 2^−∆∆Ct^ method and RPL13A as reference gene. Results are expressed as mean ± SD of at least three experiments. * *p* < 0.05 significantly different from the control (ctrl). ° *p* < 0.05, significantly different from H_2_O_2_-treated HUVECs. Ctrl (control, untreated cells); H_2_O_2_ (cells treated with hydrogen peroxide); FP (cells pretreated with fermented Pushgay); P (cells pretreated with Pushgay).

HO-1 gene expression level was determined by qRT-PCR in HUVECs treated with berries solutions (10 μg/mL) for 24 h before treatment with H_2_O_2_ for 24 h. Data showed that H_2_O_2_ induces HO-1 expression while pretreatment with FP, but not P, counteracts this effect (Figure 4).

### 3.6. Effect of Treatment with Fermented Pushgay Berries on Glutathione Reductase Activity in HUVECs

**Figure 5:**
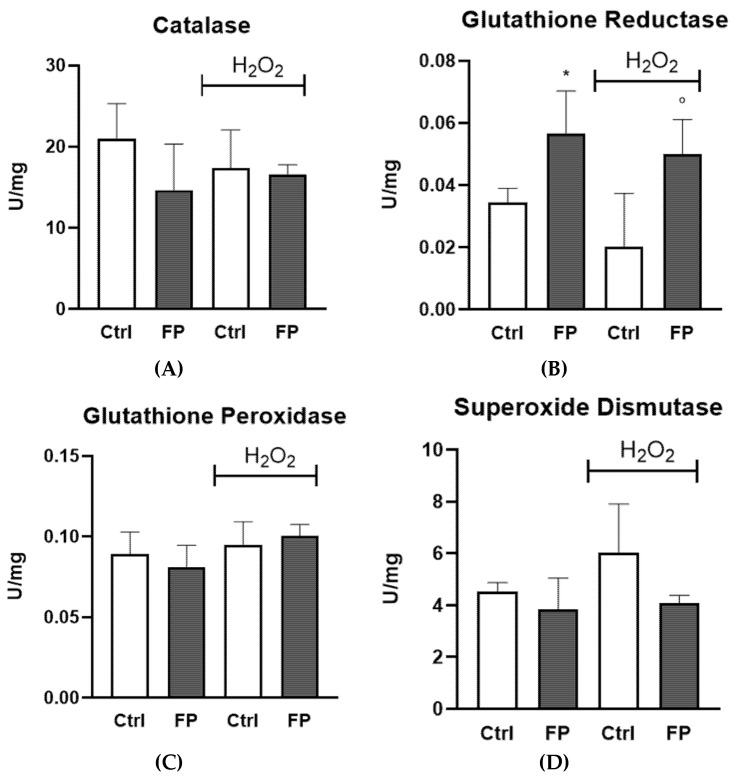
HUVECs were plated in 6-well plates (1.5 × 10^5^ cells/well) and treated with 10 μg/mL of FP for 24 h followed by treatment with H_2_O_2_ (300 µM) for 24 h. (**A**) CAT, (**B**) GR, (**C**) Gpx, and (**D**) SOD activities were assayed by the methods described in the Methods. Enzyme activity is expressed as units per milligram protein. Results are expressed as mean ± SD of at least three experiments. * *p* < 0.05 significantly different from the control (ctrl). ° *p* < 0.05, significantly different from H_2_O_2_-treated HUVECs. Ctrl (control, untreated cells); H_2_O_2_ (cells treated with hydrogen peroxide); FP (cells pretreated with fermented Pushgay).

To investigate the mechanisms underlying the antioxidant capacity of FP, glutathione reductase (GR), glutathione peroxidase (Gpx), catalase (CAT), and superoxide dismutase (SOD) activities were assayed in HUVECs in the presence or absence of H_2_O_2_. We found that FP strongly increases GR activity in HUVECs, both in the presence or absence of H_2_O_2_. No differences were observed for other enzymatic activity (Figure 5). 

### 3.7. Effect of Treatment with Pushgay Berries on RAW264.7 Cells Proliferation

**Figure 6:**
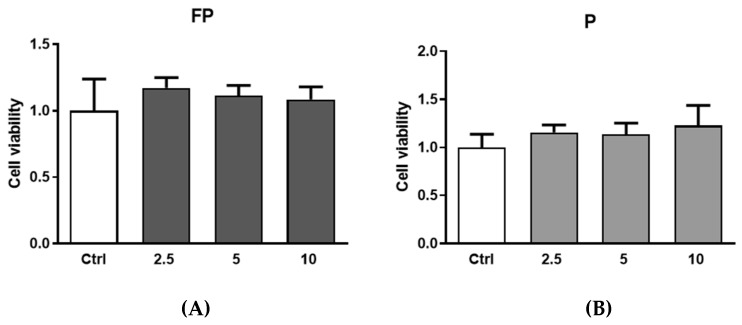
RAW 264.7 were plated in 6-well plates (3.0 × 10^5^ cells/well) and treated for 24 h with different concentration of (**A**) FP and (**B**) P solutions (2.5, 5 and 10 μg/mL). Cell viability was determined with the Burker chamber utilizing Trypan Blue as a cell dye. Results are expressed as mean ± SD of at least three experiments. ctrl (control, untreated cells); FP (cells treated with fermented Pushgay); P (cells treated with Pushgay).

Treatments of RAW 264.7 with FP and P for 24 h at different concentrations (range 2.5–10 µg/mL) were not toxic (Figure 6). Therefore, based on these experiments, we selected 10 µg/mL concentration for subsequent experiments.

### 3.8. Effect of Fermentation of Pushgay Berries on RAW264.7 Cells Growth

**Figure 7:**
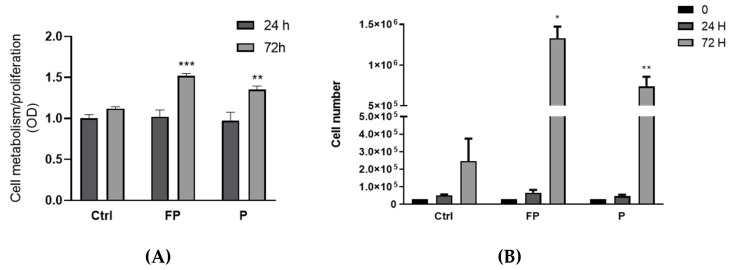
(**A**) RAW 264.7 cells were plated in 24-wells plates (3.0 × 10^4^ cells/well), treated with 10 μg/mL of FP or P and further incubated for 24 and 72 h. At the end of treatment cells were incubated at 37 ° C, 5% CO_2_ for 3 h with Alamar Blue solution. Optical density (OD) was determined with a spectrophotometer at 570 nm. (**B**) RAW 264.7 cells were seeded in 24-well plates (3.0 × 10^4^ cells/well) and treated with 10 μg/mL of FP and P and further incubated for 24 and 72 h. At the end of incubation, cell number was determined with the Burker chamber utilizing Trypan Blue as a cell dye. Results are expressed as mean ± SD of at least three experiments. * *p* < 0.05 ** *p* < 0.01 *** *p* < 0.001 significantly different from the control at 72 h (ctrl). Ctrl (control, not treated cells); FP (cells treated with fermented Pushgay); P (cells treated with Pushgay).

The effects of berries on RAW 264.7 growth were evaluated by Alamar Blue staining after treatment of cells with FP and P solutions for 24 and 72 h. We found an increase in Alamar Blue staining after 72 h of treatment with P or FP, compared to control cells, but no effect was observed after 24 h of treatment (Figure 7A). Since the Alamar Blue staining increase can indicate either stimulation of metabolism or increased cell proliferation, to discriminate between these two effects, we used Trypan Blue to assess changes in the number of cells following treatment with berries solutions for 24 and 72 h. We found a significant increase in the number of cells following 72 h of treatment with P or FP (Figure 7B), indicating stimulation of cell growth by either P and FP. 

### 3.9. Effects of Pushgay Berries on the Expression of iNOS and TNFα in RAW264.7 Cells

**Figure 8:**
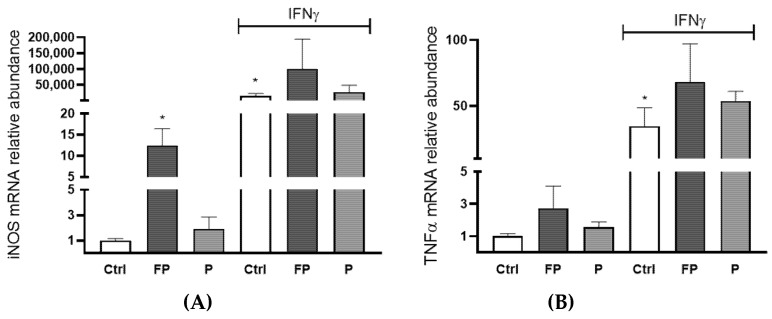
RAW 264.7 (4.0 × 10^5^/well) were seeded in six wells and incubated with 10 μg/mL of FP or P in the presence or absence of 100 U/mL of IFN-γ for 24 h. (**A**) iNOS and (**B**) TNFα genes expression was assessed using qRT-PCR analysis. Differences in gene expression were calculated using the 2^−∆∆Ct^ formula and RPL13A as reference gene. Results are expressed as mean ± SD of at least three experiments. * *p* <0.05. significantly different from the ctrl. Ctrl (control, untreated cells); FP (cells treated with fermented Pushgay); P (cells treated with Pushgay); IFN-γ (cells treated with IFNγ).

To evaluate the immunomodulatory activity of berries solutions, the mRNA levels of inducible nitric oxide synthase (iNOS) and Tumor Necrosis Factor α (TNFα) were evaluated by qRT-PCR in RAW 264.7 treated with FP or P at 10 µg/mL for 24 h in the presence or absence of Interferon γ (IFNγ) (100 U/mL). We found that treatment with FP, but not P, leads to a significant raise in the levels of iNOS mRNA in the absence of IFN-γ (Figure 8A). Treatment with P did not significantly increase TNFα expression, while FP treatment showed a slight increase, which was not statistically significant (Figure 8B). 

## 4. Discussion

The European vision of the Joint Programming Initiative “a healthy diet for a healthy life” (JPI HDHL) establishes that by 2030, all citizens will have the motivation, the ability, and the opportunity to consume a healthy diet from a variety of foods (including functional foods and supplements) to stay healthy and reduce the onset of food-related diseases. Epidemiological studies have shown that the intake of natural products, rich in antioxidants polyphenolic compounds, is related with a reduction in the risk of developing CVDs and cancer [46,47]. There is clear evidence that certain flavonoids, such as quercetin, can attenuate endothelial dysfunction [48], the first step toward atherosclerosis. Furthermore, it has been shown that bacterial strains used as probiotics, such as *Lactiplantibacillus plantarum*, may enhance the beneficial activity of phenolic compounds present in food, protecting endothelial cells from oxidative stress [49] and modulating the production of cytokines and other pro-inflammatory mediators (TNFα and iNOS) in macrophages [50]. A variety of fermented foods, such as milk, meat, fish, vegetables, cereal, and fruits, are part of human nutrition around the world, and the consumption of fermented food has been linked to reduced risk of hypertension, diabetes, obesity, and high cholesterol [51]. Variations in the antioxidant activity of fruits after lactic fermentation have often been observed, possibly due to the release of bioactive molecules from phenolic conjugated phytochemicals [52].

So far as we know, this is the first study that compares the antioxidant and immunostimulant properties of Pushgay berries before and after fermentation with *Lactiplantibacillus plantarum*. We report that treatment with Pushgay berries fermented with *Lactiplantibacillus plantarum* reduces oxidative stress induced by menadione and protects HUVECs against H_2_O_2_-induced cell toxicity more effectively than treatment with unfermented Pushgay berries. We also found that, after fermentation with *Lactiplantibacillus plantarum*, Pushgay berries promote proliferation and iNOS mRNA synthesis in mouse macrophages RAW 264.7.

Our results are in contrast with others reporting that unfermented berries, due to their high levels of polyphenolic compounds, can reduce oxidative stress [53,54,55], decrease inflammation [56], protect endothelial cells from H_2_O_2_-induced cell death [57], attenuate endothelial dysfunction, and regulate cholesterol accumulation and trafficking, along with potentially influencing gut microbiota [58]. It is possible these contrasting results are due to environmental conditions of growth or to our extraction procedures, leading to low levels of polyphenolic compounds in the non-fermented Pushgay berries under investigation in our study. Consistently with this hypothesis, it has been shown that *Lonicera caerulea* berries have a higher antioxidant capacity than blueberries because they are enriched in anthocyanins, such as anidin, peonidin, and delphinidin, which are not present in other berries [59,60]. Additionally, the content of polyphenols of *L. caerulea* may differ based on differences in the extraction approach [59]. Polyphenol contents can also be influenced by altitude [61]. Cell culture conditions used could also explain these contrasting results. It would be of interest to investigate whether treatment with non-fermented Pushgay extract has an effect on human artery endothelial cells grown in the presence of fluid shear stress to mimic the effect of blood flow, which plays a major role in endothelial cell functions [62].

As described for other berries, FP gained the ability to interfere with H_2_O_2_ production following menadione treatment [63]. Furthermore, consistent with previous studies on fermented berries [64], FP berries treatment increased HUVECs viability in the presence of H_2_O_2_, confirming that fermentation interferes with oxidative stress-induced cell damages. This is consistent with other studies that have shown that fermented blueberries have great scavenging ability against hydroxyl radicals [65,66,67]. Furthermore, we found that H_2_O_2_-treated cells, pre-treated with FP, express a lower amount of HO-1 mRNA than cells treated with H_2_O_2_ in the absence of FP. Since HO-1 synthesis is a cell response against damages caused by oxidative stress [68], these data provide more evidence that FP berries treatment reduces oxidative stress in the cell.

Although HO-1 induction promotes a protection against oxidative stress in various cell and animal models, high HO-1 levels may even sensitize the cell to oxidative stress, e.g., through the release of reactive iron [68]. In addition, it has been reported that an uncontrolled and exaggerated up-regulation of HO-1 has pro-oxidant effects [69,70,71], and thus, berries treatment could protect the cell by limiting its up-regulation.

In order to gain, at least in part, insight into the mechanism underlying the protective activity of FP, we investigated the activity of well-known antioxidant enzymes that could be stimulated by berries treatment such as CAT, SOD, and enzymes involved in the glutathione (GSH) cycle, such as Gpx and GR [72]. Our results show the specific effect of FP on GR activity but not on the other enzymes. Similarly, ginseng extract protects HUVECs against H_2_O_2_-induced damage by regulating the redox state of the cell, particularly increasing the activity of GR [73], and peptide T8 ameliorate HUVECs resistance against H_2_O_2_-induced oxidative damage by increasing GR activity [74]. Fruit-derived polyphenols may differentially affect each antioxidant enzyme, and the ability of FP to increase GR activity is of particular importance due to the critical role of GSH in endothelial function [75] and to the ability of GR in maintaining GSH supply, fundamental in the cellular control of reactive oxygen species.

Our study also showed that treatment with FP modulates innate immunity, as indicated by increased RAW 264.7 macrophages proliferation. Furthermore, treatment of these cells with FP increased basal and IFN-γ-induced levels of iNOS.

We found that Pushgay berries are enriched in quercetin and fermentation enhances their quercetin aglycone content, in line with previous data [32], and increases their intracellular antioxidant activity. Indeed, it has been previously reported that the fermentation process promotes the almost complete degradation of anthocyanins in phenolic acids, the hydrolysis of glycol-conjugates, and, in turn, higher concentrations of free flavonoids [32]. Moreover, FP exhibited higher cell-free antioxidant activity compared to the fresh berries following fermentation [32], in agreement with our results. It is known that the bioavailability of berry polyphenols is very low [8], but it is positively influenced by glucosidases during fermentation, thereby increasing in situ ROS scavenging, as well as stimulating natural antioxidant body panel [76,77]. The biotransformation of berries during the fermentation process increases the hydrolysis of phenolic compounds, thus their bioavailability [78].

Even if Hollman et al. suggested that quercetin glucoside has a higher bioavailability than the free form in ileostomized patients [79], it is plausible that the relatively high absorption of quercetin glycosides is not due to glycosylation, but rather to factors present in complex foodstuff matrices. Moreover, it would be important to investigate the role of gut microbiota in influencing the bioactivity band the permeation of dietary (poly)phenols. Indeed, the microbial bioavailability of unabsorbed bioactive compounds in the gut represents a fundamental step in the presumed bioactivity associated with (poly)phenol intake [80].

Finally, the contribution of phenolic acids in FP, which may exert an additive or synergistic effect with the quercetin aglycone within cells, must be carefully considered due to their greater bioavailability in comparison to larger phenolic molecules and their beneficial activities. More studies will be performed to clarify their possible beneficial effects in human cell models on vascular and immune system function.

## 5. Conclusions

We report that fermentation strongly increases the content of quercetin aglycone (present only in the FP, and not in P, with a concentration of 1680 ± 0.2 μg/g) and promotes the antioxidant activity of Pushgay berries. One of the possible mechanisms underlying the antioxidant activity of FP could be the increased activity of GR, an enzyme responsible for the maintenance of GSH supply, which acts as an important player in controlling the cellular levels of ROS [75]. Fermentation also enhanced the immunomodulatory properties of Pushgay berries. Our findings are in agreement with previous studies showing that fermented berries reduce oxidative stress and the levels of pro-inflammatory cytokines [81]. Overall, many studies have shown the health beneficial effects of fermented fruits. However, this evidence has been generated mainly from in vitro and in vivo studies, while clinical studies are scarce in this field. Therefore, the potential role of fermented plant products in human health must be confirmed through randomized controlled clinical trials.

## Figures and Tables

**Table 1:** Total polyphenol content and quantification of quercetin-3-O-glucoside and quercetin aglycone in lyophilized fermented or not fermented berries.

Berry	Total Polyphenol Content (μg GAE/μg Lyophilized Berry ± SD)	Quercetin-3-Oglucoside (μg/g ± SD)	Quercetin Aglycone (μg/g ± SD)
FP	2.38 ± 0.01	96.0 ± 0.1	1680.0 ± 0.2
P	2.95 ± 0.14	720.0 ± 0.1	*n.d*

SD: Standard Deviation. FP: Fermented Pushgay; P: Pushgay; *n.d*: not determined.

## Data Availability

Not applicable.
